# Residual waste management in London, England: a reality check

**DOI:** 10.1007/s10661-023-11760-2

**Published:** 2023-10-09

**Authors:** Nadia Minhas, Spyridoula Gerassimidou, Eleni Iacovidou

**Affiliations:** Division of Environmental Sciences, College of Health, Medicine and Life Sciences, Brunel University London, Uxbridge, UB8 3PH UK

**Keywords:** Residual waste, Household and non-household waste management, Waste disposal, Waste recovery, Technological lock-in, Waste management contracts

## Abstract

Residual waste is a key fraction of municipal solid waste generated, yet its management is poorly understood and has gained little attention over the past years. Using London as a case study, the present study analyses the amount of residual waste collected and managed to check on how well ahead the UK is in making progress on achieving the circular economy principles. The study found that 5 Mt of residual waste is reported to be managed in London, of which 3.5 Mt is managed via recovery operations and 1.59 Mt is managed via disposal operations. For the latter, landfills are the principal disposal option taking in 0.82 Mt of residual waste each year. Amongst the recovery options, incineration with energy recovery, is the most prevalent, accounting for the management of 1.44 Mt of residual waste. This highlights the presence of an important technological lock-in that could jeopardise UK’s ability to achieve its net zero carbon ambition. It is worth noting that the data collated and analysed depicts the movements of residual waste rather than its final treatment, pointing to blind spots in the final fate of residual waste as well as potential double counting; both of which prevent decision- and policy-making. Therefore, the need for transparency in data recording and monitoring and the creation of a level playing field for all stakeholders involved in residual waste management are of paramount importance in gradually breaking reliance on destructive treatment processes. This could empower improved segregation of waste at source, and in turn, enable the better management of residual waste. The study underlines that residual waste has a significant role to play in making the transition to a circular economy and therefore is imperative to pave the way for future policy direction on residual waste management.

**Supplementary Information:**

The online version contains supplementary material available at 10.1007/s10661-023-11760-2.

## Introduction

Residual waste, also known as black bin or black bag waste, accounts for a significant portion of the household waste generated in the UK (Defra, 2021). For the management of residual waste, the UK has been relying on landfills for many years, with 50.8 million tonnes of waste disposed into landfill in 2018 (Defra, 2022a). The introduction of the Landfill Tax in 1996 (i.e. tax on the waste that enters a licensed landfill at a standard rate of £98.60 per tonne of waste disposed to landfills as of April, 2022; HMRC, 2022) and the Waste Framework Directive (Directive 2008/98/EC, (2008) prompted progress towards diverting waste from landfills (Defra, 2010; HM Government, 2018a). However, as emphasised in the 25-Year Environment Plan (25YEP) (HM Government, 2018b) and the Resources and Waste Strategy England (HM Government, 2018a), there is still a need to reduce residual waste and to transition away from linear practices via the introduction of alternative waste management options for residual waste (HM Government, 2018a, b). This has been seen as an important drive to empower changes in residual waste management.

According to the 25YEP, residual waste is defined as ‘waste that cannot be reused or recycled’ (HM Government, 2018b, pg. 94); a definition that is open to misinterpretation, impairing any attempts for improvements in residual waste management. A similar definition is provided in the Resources and Waste Strategy (HM Government, 2018a), where residual waste ‘is the mixed material that is typically incinerated for energy recovery or landfilled. Much of the products and materials contained in this waste could have been prevented, reused or recycled’ (HM Government, 2018a, pg. 137). In other governmental reports, residual waste is termed as ‘waste from households’ regular collections (black bags), bulky waste, residual waste from civic amenity centres, and rejects from recycling’ (Defra, 2021, pg.12). Environmental Improvement Plan defines residual waste as ‘waste that is sent to landfill, put through incineration or used in energy recovery in the UK, or that is sent overseas to be used in energy recovery’ (HM Government, 2023a, pg.144). Moreover, the Environment Act 2021 lacks emphasis on residual waste and steers away from defining the term. Similarly, at the EU level, the definition of residual waste remains unclear. And yet, for instigating improvements in residual waste management, a mutual agreement on residual waste definition is of great importance, as the lack of it could lead to fragmented and inefficient efforts to deal with this waste stream, as highlighted by Malinauskaite et al. (2017).

The lack of evidence on the fate of residual waste in the UK creates an additional strain in promoting sustainability and circularity in the system, especially as the UK has introduced a target to reduce residual waste by 50% by 2042, set out in the Environmental Improvement Plan (Defra, 2022b; HM Government, 2023a). The London Waste and Recycling Board (LWARB) pointed out that the starting point of moving towards a green circular economy is focusing on improvements at big urban centres (LWARB, 2017). Therefore, this study, using London as a case study, analyses the amount of residual waste collected and managed, with a view to providing a reality check on how well ahead the UK is in making progress on implementing circular economy principles. London is the UK’s capital city and one of the nine regions under the English administration with the biggest and most congested populated area with an estimated population reaching 8.7 million in 2021, making London a complex and interesting landscape for residual waste management (Greater London Authority, 2022).

The study begins by providing insights into the administrative levels and responsibilities of waste management in London’s local government and then it analyses data to decipher the amount of residual waste collected and managed using Local Authority (LA) and waste management operators reporting databases. The study then attempts to map the fate of across different waste management options and make sense of progress with waste management objectives and targets in line with the waste hierarchy. Lastly, the study unpacks the underlying factors associated with residual waste management in London and makes recommendations for where interventions are needed that could potentially promote efficiency and sustainability in the waste management sector.

## Background

London is composed of 32 boroughs, and the City of London, which is not itself a borough but has its own authority; a list of all London boroughs is provided in *Supplementary Material*, Table S1, along with population data. Waste management practices vary across the 32 boroughs and the City of London, and are based on an administrative structure, depending on whether the boroughs are responsible for either the collection or the collection and management of their waste. These are known as (1) single-tier authorities, commonly known as unitary authorities—responsible for both the collection and management of their waste (Parliament, n.d.); and (2) two-tier authorities—responsible for the collection of their waste, typically known as waste collection authorities (WCA).

**Fig. 1:**
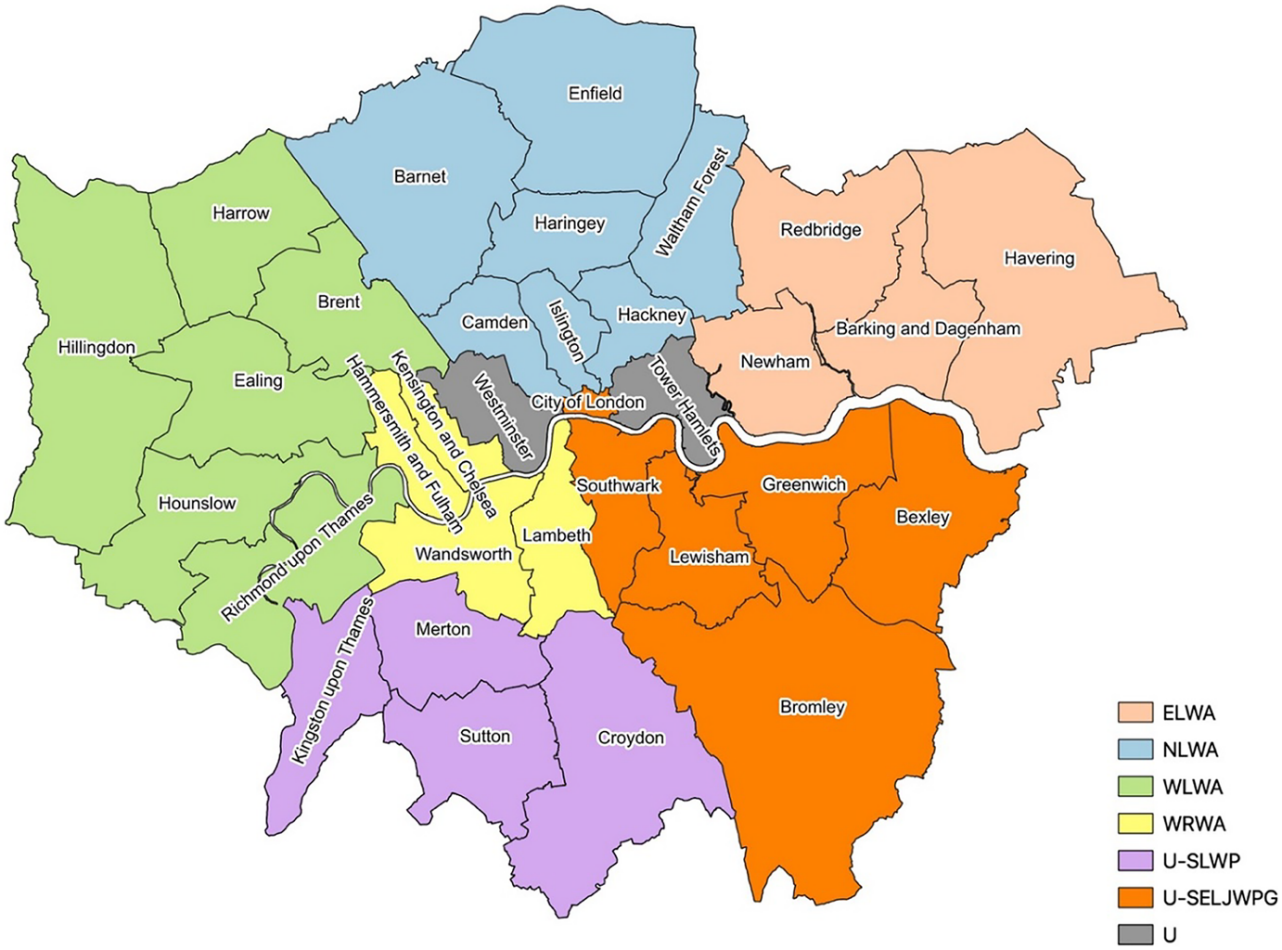
London’s waste management administrative structure into waste disposal authorities (WDA) and unitary authorities (U). ELWA (peach): East London Waste Authority; NLWA (blue): North London Waste Authority; WLWA (green): West London Waste Authority; WRWA (yellow): Western Riverside Waste Authority. Unitary authorities include U-SLWP (purple): South London Waste Partnership; U-SELJWPG (orange): South East London Joint Waste Planning Group; U- (grey) Unitary authorities, that do not belong to a non-statutory group/partnership

Two-tier authorities usually work collaboratively with other authorities to form a waste disposal authority (WDA) that is responsible for the disposal/management of their waste. Similarly, unitary authorities may form partnerships to organise the management of their waste. Figure 1 shows the organisation of London boroughs into two-tier and unitary authorities according to the waste management responsibilities.

As seen in Fig. 1, there are four WDAs and 12 unitary authorities in London. SLWP, illustrated with purple in Fig. 1, is a voluntary partnership between the unitary authorities of Croydon, Kingston Upon Thames, Merton and Sutton, which provide waste collection and management services to 1 million residents (SLWP, n.d.). The Joint Waste Committee is responsible for making the key decisions for this partnership (London Council, n.d.). Additionally, Southwark, Greenwich, Lewisham, Bexley, Bromley and the City of London are all unitary authorities that formed a unique joint waste planning group called the South East London Joint Waste Planning Group (SELJWPG) (London Borough of Bexley, 2022). Tower Hamlets and Westminster are not part of any voluntary waste partnership.

## Methodology

The study followed a two-step methodology; 1 – focusing on data collection and analysis and 2 – reflecting and interpreting data analysed from a policy perspective.

**Step one**: Data for each of the 32 London boroughs were retrieved from the Waste Data Interrogator (WDI) from the year 2020 (WDI, 2022), using the tonnage reported as ‘waste received’ available from the Department for Environment, Food and Rural Affairs (Defra). The dataset used is version 4, which was published in February 2022. The WDI dataset includes the amount and destination of residual waste across all 32 London boroughs, including also data reported under ‘London’, ‘Greater London’ and ‘Central London’ regions. The reporting under these regions is a remnant of old reporting practices still in use by waste management operators and is hereafter called the ‘London variations’. The data was filtered to extract/refine data related to the residual waste reported in London using the ‘Recorded origin’ column. London was selected as the ‘origin region’. Then, the Substance-Oriented Classification (SOC) category (10-mixed ordinary waste) and the SOC subcategory (Household and similar wastes) were selected as in the European Waste Classification for Statistics (EWC-Stat) categories (Eurostat, 2010), following the advice of professionals from the Environment Agency (EA) and Defra. In the ‘Basic waste category’, Household/Industrial/Commercial (Hhold/Ind/Com) was chosen.

Using the List of Waste, code 20-Municipal Waste was selected following the waste categorisation used in England (UK Legislation, 2005). Within this code, the data selected was under code 20 03 Other municipal wastes, specifically 20 03 01 (Mixed municipal waste), 20 03 03 (Street-cleaning residues), 20 03 07 (Bulky waste), 20 03 99 (Municipal wastes not otherwise specified) and 20 03 02 (Waste from markets), selected in accordance to the EU guidance on the classification of waste that describes the household waste and waste similar to household waste, the equivalent of Local Authority Collected Municipal Waste (LACMW) in the UK (Eurostat, 2010). The selection was validated by the EA professionals. Liquids and sludges which are usually reported under 20 03 were excluded from the analysis.

After filtering the data, the destination of residual waste in each borough, including the London variations were extracted and analysed using the disposal (D) and recovery (R) codes (Waste Framework Directive—Annexe I and II). In the D and R classification, waste treatment options are assigned a four or six-digit code; the first two digits describe the main treatment option, and the next two or four digits specify the waste treatment details (see Table S2 in *Supplementary Material*). Here, the residual waste fate reported under D and R options is grouped into the two-digit code processes to cut through complexity and provide a rounded view of the residual waste relating to the major recovery and disposal processes. The coefficient of variation (CV) was calculated to estimate the variation among boroughs of the same authority as well as the variation among boroughs of the wider level of WDAs and unitary authority.

To validate the data analysis and identify potential discrepancies, Waste Data Flow (WDF) data from Jan 2020 and Dec 2020 were retrieved, analysed and compared against the WDI data. WDF is the database that local authorities use to report their collected waste without providing details on the fate of waste. From the WDF, the ‘Total collected residual waste’ report was selected for London which includes all the boroughs and the WDA reporting. Then, the ‘Household residual waste’ and the ‘non-Household residual waste’ data were selected to match the selection of data from the WDI and reflect the LACMW (WDF, 2014). A Pearson correlation analysis was carried out to assess the linear relationship between WDI and WDF including also hypothesis test to determine the significance of correlation with a significance level of 0.05.

**Step two**: This step looks into the waste management contracts between London boroughs and waste management companies by using data on ‘Waste contracts register- Contract Expiry dates’ available from the London Datastore by Greater London Authority (GLA). Disposal contracts and their duration and extension available were collected. Contract data was merged with each borough’s major treatment method by the highest tonnage associated with each operation, expressed as a percentage, to understand the relationship between stakeholders, local authorities and waste management providers within London.

## Results and discussion

### Residual waste fate in London

The data extracted and analysed from the WDI provides insights into the movement of residual waste along the waste management chain; hence, this data should not be interpreted as residual waste generation. Of the 5.08 Mt of residual waste reported in the WDI, 3.49 Mt is sent to recovery operations, whereas 1.59 Mt of residual waste is sent to disposal operations. A list of the dominant waste disposal and recovery operations using the two-digit code, which was selected for data presentation and interpretation, is as follows (a detailed list of all processes included under each two-digit process can be seen in *Supplementary Material*).

Disposal operations: **D01—**deposit into or onto land (e.g. landfill); **D05—**specially engineered landfill; **D08—**biological treatment; **D09—**physicochemical treatment; **D10—**incineration on land; **D13—**blending or mixing prior to submission to any of the operations numbered D01 to D12; **D14—**repackaging prior to submission to any of the operations numbered D01 to D13; **D15—**storage pending any of the operations numbered D1 to D14.

Recovery operations: **R01—**incineration of waste for use principally as a fuel or other means to generate energy; **R03—**recycling/reclamation of organic substances which are not used as solvents; **R04—**recycling/reclamation of metals and metal compounds; **R05—**recycling/reclamation of other inorganic materials; **R12—**exchange of waste for submission to any of the operations numbered R01 to R11; **R13—**storage of waste pending any of the operations numbered R1 to R12.

**Fig. 2:**
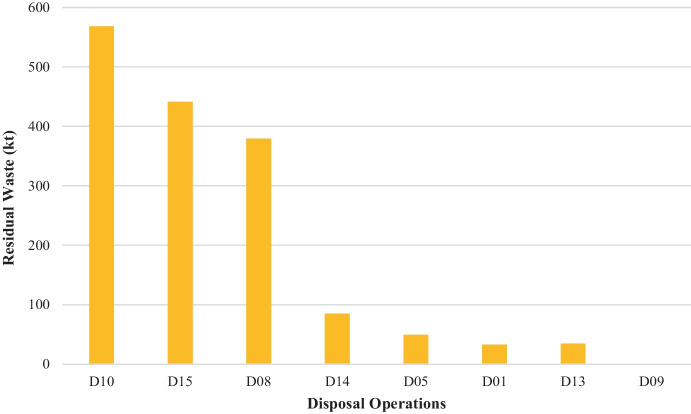
Tonnage (kt) residual waste sent to disposal operations (D codes), including the tonnage reported under the London variations as reported in the WDI (WDI, 2022)

Figure 2 shows the proportional distribution of residual waste, to waste treatment options under the D code. The dominant disposal operation for residual waste is D10 taking up 568 kt of residual waste. The second and third largest disposal options are D15 and D08, at 441 kt and 379.3 kt, respectively.

D15, D14 and D13 are intermediary waste treatment options accounting for the uptake of a total of 560.5 kt tonnes of waste. However, they do not provide insights into the final sink of residual waste. For one to be able to identify the final sink, one should be able to track residual waste as they move along the waste management chain. This highlights that using data solely for WDI is not sufficient to provide insights into the final fate of all residual waste. Furthermore, the treatment via D08 could refer to mechanical biological treatment (MBT) in which output is contaminated and needs to be disposed of. This also accounts for an intermediary stage rather than a final sink and points to further implications when using this data to understand the final fate of residual waste.

Similar data inconsistencies are recorded in the recovery operations as well. The R13 and R12 represent temporary storage of waste or a transfer activity, the latter including some kind of pre-treatment prior to exchange, or submission, to other operations for recovery. Both options capture the logistical processes of handling and pre-sorting of waste instead of its final treatment. This creates blind spots in the actual final fate of residual waste, which as opposed to disposal operations, where waste most likely moves into treatment activities within the disposal classification; the residual waste that is reported under recovery might end up moving to treatment options within the disposal classification.

**Fig. 3:**
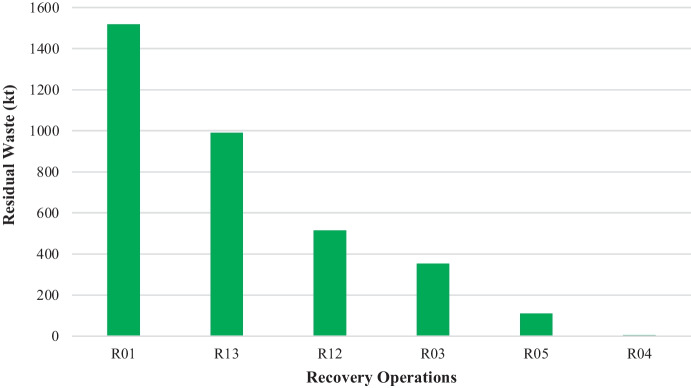
Tonnage (kt) of residual waste sent to recovery operations (R codes), including tonnage reported under the London variations as reported in the WDI (WDI, 2022)

Figure 3 presents the distribution of residual waste to the major recovery operations. R01 is the dominant treatment option taking up 1.5 Mt of residual waste. This option could be the final sink of residual waste. It must be noted that four-digit processes under the R01 code (see Table S2 in *Supplementary Material*) hint at the fact that there might be residues from other treatment processes entering this treatment option (e.g. R01.03 represents 10 kt of stabilised residual waste in the form of refuse-derived fuel (RDF)) underlining potential duplication of data across the R codes arising from residual waste movements.

The second and third largest recovery routes for residual waste treatment are R13 and R12, accounting for 991 kt and 515 kt of residual waste, respectively (Fig. 3). These are followed by R03 which is very wide and covers many activities, primarily the recovery of organic material from residual waste possibly via composting or anaerobic digestion at the mechanical biological treatment (MBT) facilities. Based on our analysis, the R03 accounts for the fate of 352 kt of residual waste in total, distributed in various sub-categories, namely R03.01 (222 kt) which is bulking up the organic waste fraction, R03.03 (6 kt) that is mechanical reprocessing, R03.04.03 (28 kt) which is the production of RDF through mechanical processing and R03.06 (1.8 kt) that is mechanical physical stabilisation (see further details in Fig. S5.2 in *Supplementary Material*). The R04 and R05, which are also wide in nature and cover a lot of activities (see Figs. S5.3 and S5.4 in *Supplementary Material*), are according to WDI data the destination of 4.3 kt and 111 kt of residual waste, respectively. Both of these codes are likely associated with sorting processes that occur at MBT facilities to remove recyclable waste materials that enter the recycling chain for their end treatment.

To summarise, of the 5.08 Mt of residual waste reported in London, 40% is sent to incineration (with/without energy recovery: D10 and R01) totalling up to 2.08 Mt. This aligns with the Defra data on LACW treatment, which report that around 2.2 Mt is incinerated (Defra, 2021). The second treatment option of residual waste as reported in the WDI is the intermediary treatment stage of storage/transfer of residual waste (D13, D14, D15, R13, R12) at 2.06 Mt, which creates blind spots in the interpretation of data as it is not possible to map the final fate of residual waste moving along the waste management chain. This highlights the need for a transparent reporting system on the amount and types of waste that enter recovery and disposal processes and that informs the final fate of residual waste so that progress towards its reduction and sustainable management can be made.

Only 6% of the residual waste reported in the WDI is being disposed of in the landfill, as represented by the D01 (non-hazardous landfill) and D05 (specially engineered landfill) codes with 32.9 kt and 49.3 kt, totalling 82.2 kt being landfilled. This estimate is double the amount of LACW being sent to the landfill as reported by Defra which is at 49 kt (Defra, 2021). The diversion of residual waste from landfill to other treatment options could be attributed to the implementation of the Landfill Directive and the UK Landfill Tax (Defra, 2010; HMRC, 2022) and has contributed to a reduction of waste carbon emissions by 5% between 2019 and 2020. While diverting residual waste from landfill may be construed as positive progress towards eliminating the disposal of waste, it has created a reliance on incineration operations. The most recent WRAP report on gate fees suggests that there was a small reduction in gate fees for WtE facilities due to new proposed plants in the pipeline, hence increasing competition. The gate fee for WtE comes up to £150, which is 20% less than the gate fees for landfill—£187 (WRAP, 2021).

While incineration is an attractive alternative to landfill and an established process for waste management providers that can create revenue from selling the energy produced (WRAP, 2021), it remains a debatable waste management option as it has reportedly led to a 3% increase in carbon emissions since 2019 (Climate Change Committee, 2022). This has alarmed the UK government, as the increased use of incineration plants has now become a major hurdle to the UK government’s ambition to reduce greenhouse gas emissions by 78% by 2035 to meet its net zero targets (HM Government, 2021). However, incineration is not included in the UK Emissions Trading Scheme (ETS), a cap and trade scheme that came into effect on January 1, 2021, which seeks to limit greenhouse gas (GHG) emissions from energy-intensive industries to achieve decarbonisation ambitions (HM Government, 2023b). A cap is set on the total amount of greenhouse gases that can be emitted (which decreases over time) as a way to achieve the Net Zero 2050 targets and other legally binding carbon reduction commitments.

The reason for incineration not being included in the ETS is multi-faceted and involves the strong opposition of the local government. Local government has grown a dependence on incineration facilities and therefore, their inclusion in the UK ETS would make it a costly process, as it will be challenging for some facilities—due to their size, age, location and financial status—to invest in carbon reduction/capture technologies to comply with the ETS scheme. Moreover, with high landfill gate fees, the prospect of incineration becoming economically unattractive creates an enormous burden for local governments to identify new ways to manage their residual waste.

### Variations in the fate of residual waste across London boroughs

**Fig. 4:**
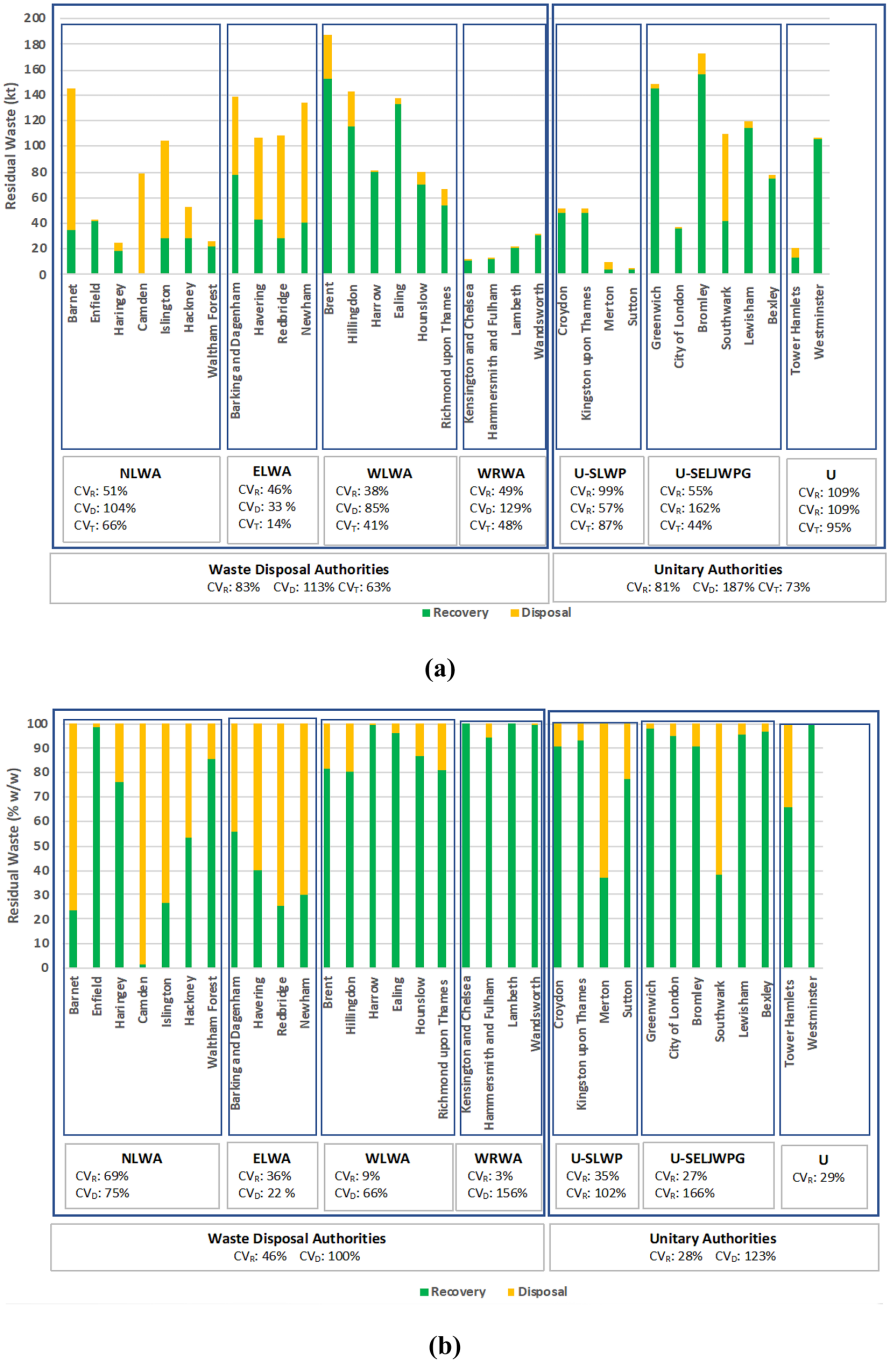
**a** Total residual waste (kt) treated via recovery and disposal activities across all 32 London boroughs grouped by authority; **b** percentage contribution of recovery and disposal activities based on the total amount of residual weight treated grouped by authority; both figures exclude tonnage reported under ‘London’, ‘Central London’ and ‘Greater London’ to avoid data double counting. CV_R_: coefficient of variation of recovery; CV_D_: coefficient of variation of disposal; CV_T_: coefficient of variation of total residual waste treated

Figure 4 depicts the amount of residual waste sent to disposal and recovery operations, and the percentage contribution of each operation to the management of residual waste. The highest amount of residual waste reported in the WDI was from the borough of Brent at 186.8 kt, followed by Bromley at 172.8 kt, and Greenwich at 148.8 kt. The residual waste reported under all other boroughs ranged from 145 to 20 kt; the lowest amount reported was from the boroughs of Hammersmith and Fulham and Kensington and Chelsea at a range of 13 to 10 kt, and at Merton and Sutton at 10.34 kt and 4.69 kt, respectively. The highest amount of residual waste recorded entering disposal activity is waste from Barnet at 111kt.

Figure 4a shows the CV for both recovery (CV_R_) and disposal (CV_D_) operations in order to obtain insights on the variability of the amounts of residual waste treated among boroughs of the same authority, whether WDA or unitary. For WDAs, the CV_R_ fluctuates between 38 and 51%, while for unitary authorities, CV_R_ ranges at a higher level from 55 to 109%. This indicates that the amount of residual waste that is recovered from unitary authorities is more variable among boroughs compared to the amount that is recovered from WDAs. However, the CV_D_ exceeds 85% for most authorities, except ELWA and U-SLWP, indicating that the amount of residual waste that is disposed from authorities is highly variable among authorities. Only ELWA presents a relatively low variability among boroughs for both operations (CV_R_: 46% and CV_D_: 33%) implying that the amounts of residual waste that are treated through recovery and disposal options are relatively uniform. In addition, the total variations (CV_T_) show a relatively lower variability of total amounts of residual waste that are treated among boroughs of the same authority compared to CV_R_ and CV_D_. This indicates that the total amount of residual waste that is treated is relatively more uniform among boroughs of the same authority, while there is higher variability in terms of residual waste amounts that are recovered and particularly in amounts that are disposed of. By comparing the CVs between WDAs and unitary authorities, there are no clear differences indicating that the variability of boroughs from unitary authorities does not considerably differ from this from WDAs. The CV calculated for 33 boroughs showed also high variability (CV_R_: 82%; CV_D_: 135%; and CV_T_: 66%).

Figure 4b shows the CVs in order to obtain insights on the variability of residual waste management in terms of the disposal and recovery contribution among boroughs of the same authority. In most authorities, the CV_R_ is less than 36% showing that the recovery treatment option has a relatively homogeneous contribution to residual waste management among boroughs of the same authority. The only exception is NLWA (CV_R_: 69%) due to the high recovery of Enfield (98.5% w/w) and the high disposal of Camden (98.2% w/w). For disposal contribution, the variability fluctuates at higher levels due to its lower contribution to residual waste management compared to recovery for most boroughs, which in turn inflates the CV_D_. Moreover, the variability among boroughs of WDA is higher (CV_R_: 46%) than that of unitary authorities (CV_R_: 28%). This is due to the relatively lower contribution of recovery and, consequently, the higher contribution of disposal in boroughs from WDAs compared to unitary authorities. The CV calculated for 33 boroughs was 40% and 108% for recovery and disposal, respectively.

**Fig. 5:**
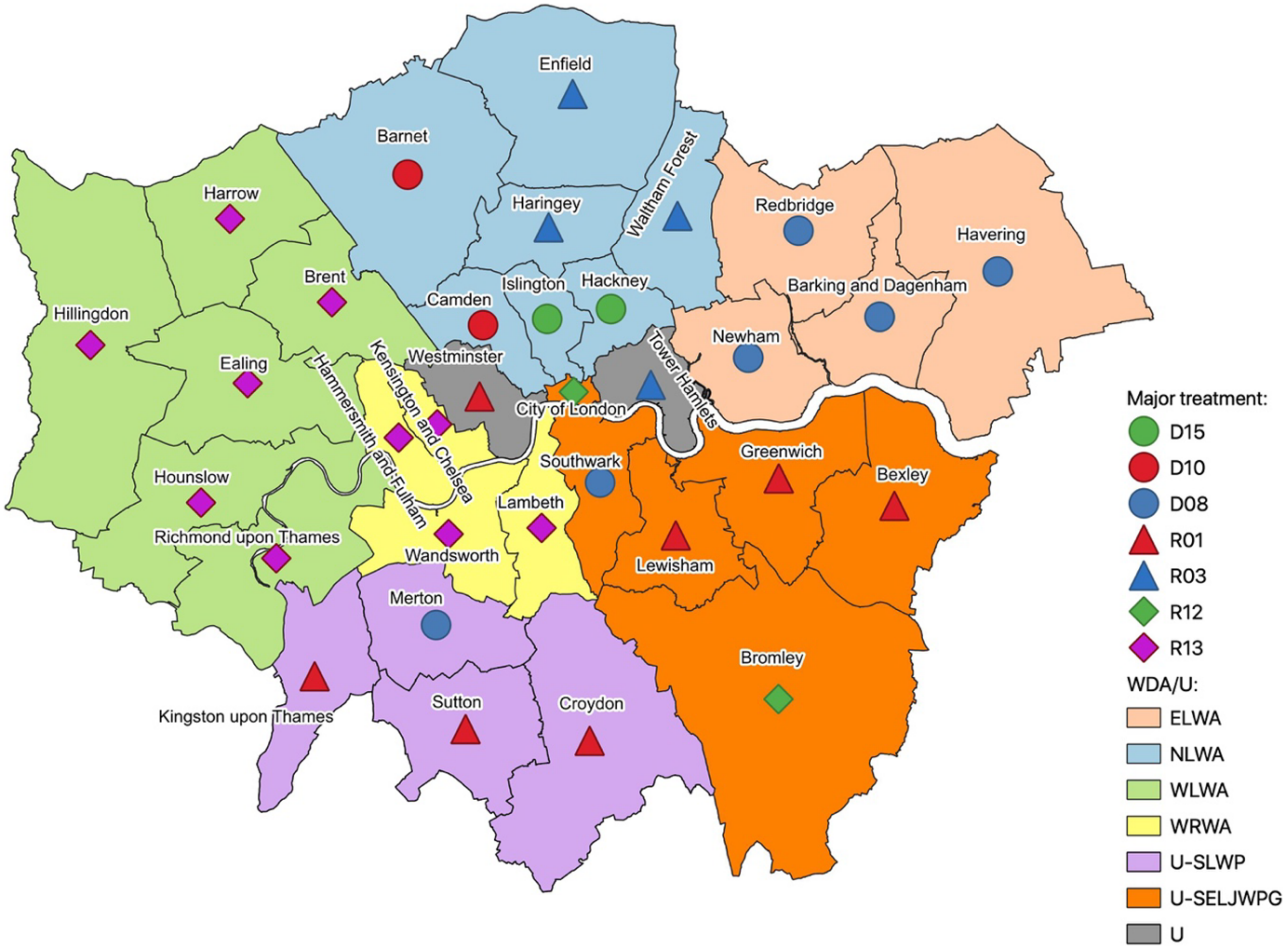
Major waste management treatment options for the management of residual waste across all London boroughs

It must be noted that Fig. 4(a, b) excludes the tonnage reported under the London variations. The tonnage reported under these regions can be found in the *Supplementary material*. It is worth noting that within the WDI, 13.16 tonnes were reported as other fates, i.e. OF (other fate), NS (not specified) and did not correspond with a D and R code; hence, this tonnage has been excluded from the overall total. Figure 5 provides insights into the major treatment technology used by each London borough for the treatment of their residual waste.

Seven boroughs use incineration with energy recovery as their main residual waste treatment option, and only two boroughs use incineration without energy recovery (a disposal option), namely Barnet and Camden boroughs. Ten boroughs appear to be sending their residual waste to temporary storage options with unknown final fate, and eight boroughs use recovery as the major treatment option for their residual waste. The pervasiveness of the treatment option depends on the contracts that local authorities have with waste management providers, analysed in the ‘Waste management contracts’ Section.

It must be noted that using the D and R codes to report the fate of residual waste is hindering progress in pushing residual waste management up in the waste hierarchy. Ultimately, this hampers any efforts to promote circularity, as waste resource value is lost in the waste management chain. The lack of insights on the composition of residual waste implies that valuable items may enter this waste stream, that could have been repaired or reused. Specifically, a recent study suggests that waste electrical and electronic equipment (WEEE) is often disposed of in the residual waste stream. However, this is highly valuable waste that should be diverted to reuse and recycling processes. The same study suggests that if WEEE items were recovered from the residual waste stream, £ 196–215 million of potential monetary value could be recouped by 2030 (Pekarkova et al., 2021).

### WDI vs WDF

According to WDF, around 4.08 Mt of residual waste was collected in London in 2020, highlighting a 1 Mt discrepancy between WDF and WDI datasets. This, as previously mentioned, could be a result of double counting in the WDI due to the reporting of waste under different treatment codes when it moves along the waste management chain. Another difference between WDI and WDF is that the former provides details on the destination of residual waste reported under code 20 in the List of Wastes that are used to record municipal waste streams and is often likely to include also construction, demolition and excavation waste (CDEW), and involves the movement of waste from one treatment stage to another. Therefore, the data on WDI is likely to be higher than the data recorded in WDF. In the WDF, two data strings are reported: (1) household waste and (2) non-household waste. These definitions have since 2020 changed and data on WDF are now reported under waste from households (WfH) and waste not from households (WnfH). The data used herein are from 2020 and refer to household and non-household waste that corresponds to residual waste. The residual fraction of household waste includes regular waste, other and bulky waste collected from households and household waste from civic amenity sites. The non-household residual waste includes non-household waste from civic amenity sites, highways waste, construction and demolition (C&D) waste (other than that recorded in the CDEW, category), grounds waste (other than that recorded in the C&D category), commercial and industrial waste (other than that recorded under the specified categories), beach cleansing, fly-tipped waste clearance and other collected waste. To the best of the authors’ knowledge, some of the aforementioned categories may be reported in the WDI under code-20 Municipal waste, and thus included in the analysis, and others may not, as there is no clarity on what is reported under codes 20 03 0X. Moreover, the small tonnage recorded under the non-household category in the WDF denotes that this may be the municipal fraction of residual waste that is not from households. Therefore, to allow for a holistic representation of data, Table 1 illustrates the amount of both household and non-household residual waste collected in the region of London as reported in the WDF.

**Table 1:** WDF reporting data on ‘Total Collected Residual Waste’ in 2020

***WDF reporting***	***Total household residual waste*** ***(kt)***	***Total non-household residual waste*** ***(kt)***	***Total collected residual waste combined*** ***(kt)***
*32 London boroughs (including the City of London)*	1919.6	522.7	2442.2
*WDA*	1338.8	299.2	1638.1
*Unitary authorities*	638	182.3	820.3
*WDA and unitary authorities*	1976.8	481.6	2458.4

As shown, in Table 1, the sum of the total amount of collected residual waste from each borough appears to be lower (ca. 57 kt) for household waste and higher for non-household residual waste (ca. 41 kt) when compared to the WDA and unitary authorities; the total difference of household and non-household waste combined is nearly a 16 kt. An explanation for the difference in the reported tonnage could be that the WDA report also the waste collected at the civic amenity sites and household waste recycling centres (HWRC) that take up the waste that may not be recyclable, such as in the NLWA’s bulky waste recycling centres to dispose of their residual waste. Facilities like these are run by the London Energy company for NLWA (NLWA, n.d.). This would mean boroughs alone collect slightly less residual waste than the WDA itself, which may be reflected in reporting.

**Fig. 6:**
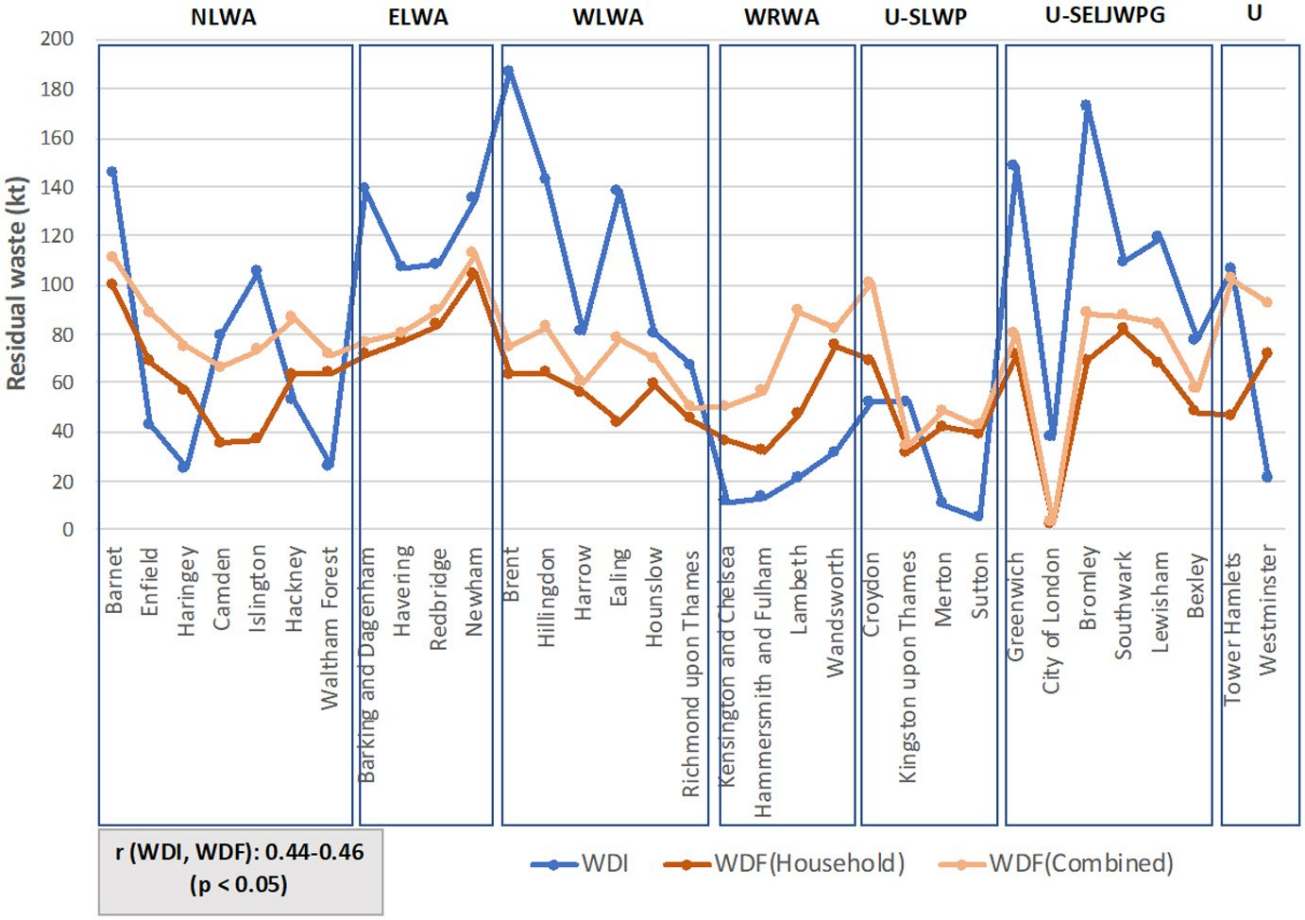
Comparison of WDI and WDF total tonnage of residual waste (kt) across all 32 London boroughs. WDF combined means—household waste and non-household waste summed together

Figure 6 presents the data on residual waste from the two databases, WDI and WDF including data on both household residual waste and household and non-household data combined, for inclusivity and robustness. The figure shows that generally there is alignment in the data between the two datasets, across all 32 London boroughs and in the majority of boroughs, the data recorded on the WDF appears lower than what is reported in the WDI which is expected given the data discrepancies in the WDI. Specifically, the data that appears to be higher in the WDI than in the WDF could be due to (1) including waste streams reported under the municipal waste classification (e.g. CDEW) and (2) double-counting when reporting the destination and final sink of residual waste when using the R&D codes.

Pearson’s correlation analysis showed that WDI has a moderate positive correlation with WDF data on both household (0.47) and combined (0.44) residual waste, while the hypothesis test showed that this linear relationship is significant (*p* < 0.05). The correlation is moderate, as previously mentioned, due to discrepancies arising from different data reporting methods. The cases where WDF data is higher than in the WDI could be the result of the data reporting under the London variations instead of under a specific borough in the WDI.

### Waste management contracts

In the UK, the leading waste management companies by revenue in the year 2021 are Veolia Environmental Services, Biffa Group, Suez Recycling and Recovery, Viridor FCC Environment, DS Smith Recycling and Renewi (Statista, 2023). In London, the main waste management services (excluding collection) providers are London Energy, Renewi, Suez, Viridor, Veolia and Cory Environment, providing services to 7, 4, 6, 11, 4 and 6 boroughs respectively.

Table 2 provides further details on the major services, i.e. management of more than 50% of residual waste, provided to each London borough, categorised by type of authority (i.e. WDA and unitary) providing D15 and R03 treatment activities with contracts ending in 2025. WLWA’s major providers are Suez and Viridor providing R13 treatment activities with contracts running until the end of 2041 and 2034, respectively. WRWA’s residual waste provider is Cory Environmental, with a contract ending in 2032. Cory Environmental promotes R13 as the major management option for all boroughs, acting as such as intermediaries in the waste management chain. The unitary authorities have contracts with various providers and are not as uniform as for WDAs (Table 2). For example, all boroughs within SLWP (i.e. Croydon, Kingston upon Thames, Merton and Sutton) have R01-Incineration with energy recovery contracts with Viridor until 2043. SELJWPG, the unitary waste planning group, has a varied portfolio of contracts with waste management companies.

**Table 2:** London boroughs’ major waste management method including contract duration with the major waste management provider. Additionally, this shows the major waste disposal contracts that each WDA has and the percentage contribution to their total residual waste management

***WDA***	***Borough name***	***Provider***	***Contract duration***	***Major treatment (D/R)***	***Total RW (kt)***	***Total RW managed by the major treatment option (as in column 5)***
*NLWA*	Barnet	London Energy	2025	D10	146 kt	60% (88 kt)
*NLWA*	Enfield	London Energy	2025	R03	43 kt	87% (37 kt)
*NLWA*	Haringey	London Energy	2025	R03	25 kt	75% (19 kt)
*NLWA*	Camden	London Energy	2025	D10	79 kt	62% (50 kt)
*NLWA*	Islington	London Energy	2025	D15	105 kt	73% (76 kt)
*NLWA*	Hackney	London Energy	2025	D15	53 kt	46% (24 kt)
*NLWA*	Waltham Forest	London Energy	2025	R03	26 kt	67% (17 kt)
*ELWA*	Barking and Dagenham	Renewi	2027	D08	139 kt	44% (61 kt)
*ELWA*	Havering	Renewi	2027	D08	107 kt	60% (64 kt)
*ELWA*	Redbridge	Renewi	2027	D08	108 kt	72% (78 kt)
*ELWA*	Newham	Renewi	2027	D08	134 kt	68% (92 kt)
*WLWA*	Brent	Suez/Viridor	2041/2034	R13	187 kt	34% (64 kt)
*WLWA*	Hillingdon	Suez/Viridor	2041/2034	R13	143 kt	34% (48 kt)
*WLWA*	Harrow	Suez/Viridor	2041/2034	R13	80kt	74% (60 kt)
*WLWA*	Ealing	Suez/Viridor	2041/2034	R13	138 kt	58% (80 kt)
*WLWA*	Hounslow	Suez/Viridor	2041/2034	R13	80 kt	85% (68 kt)
*WLWA*	Richmond Upon Thames	Suez/Viridor	2041/2034	R13	67 kt	66% (44 kt)
*WRWA*	Kensington and Chelsea	Cory Environmental	2032	R13	11 kt	100% (11 kt)
*WRWA*	Hammersmith and Fulham	Cory Environmental	2032	R13	13 kt	93% (12kt)
*WRWA*	Lambeth	Cory Environmental	2032	R13	21 kt	99% (21 kt)
*WRWA*	Wandsworth	Cory Environmental	2032	R13	32 kt	85% (27 kt)
*U-SLWP*	Croydon	Viridor	2043	R01	52 kt	81% (42 kt)
*U-SLWP*	Kingston Upon Thames	Viridor	2043	R01	52 kt	76% (40 kt)
*U-SLWP*	Merton	Viridor	2043	D08	10 kt	41% (42 kt)
*U-SLWP*	Sutton	Viridor	2043	R01	4.6kt	67% (3.1 kt)
*U- SELJWPG*	Greenwich	Veolia/SELCHP	2028*	R01	149 kt	43% (65 kt)
*U- SELJWPG*	City of London	Cory Environmental	2027	R12	37 kt	93% (35 kt)
*U- SELJWPG*	Bromley	Veolia	2027(+ 8)	R12	173 kt	47% (82 kt)
*U- SELJWPG*	Southwark	Veolia	2033	D08	109 kt	62% (68 kt)
*U- SELJWPG*	Lewisham	Viridor	2027	R01	119 kt	69% (82 kt)
*U- SELJWPG*	Bexley	Cory Energy	2047	R01	77 kt	73% (56 kt)
*U*	Tower Hamlet	Cory Environmental		R03	21 kt	46% (9.6 kt)
*U*	Westminster	Veolia/SELCHP	2023	R01	106 kt	85% (90 kt)
*N/A*	London	N/A	N/A	R01	1.8 Mt	51% (965 kt)
*N/A*	Greater London	N/A	N/A	D15	545 kt	40% (220 kt)
*N/A*	Central London	N/A	N/A	R01	1.3 kt	100% (1.3 kt)

*Contract between Greenwich and SELCHP was to end in 2023, however announced in early 2022 that the contract will be extended for another 5 years (Royal Borough of Greenwich, 2022)

As shown in Table 2, WtE has become an attractive management option for local authorities as it is an easy-to-operate, well-established option, with low behaviour change demands from the public (Climate Change Committee, 2022). This points to a technological lock-in that is likely to hinder the UK’s ability to improve resource recovery, not to mention the 78% reduction in its net zero carbon emissions up until 2033 (HM Government, 2021); especially, as contracts with waste management companies for WtE facilities range from 5 to 24 years. As shown in Table 2, 19% of contracts is with WtE providers that last up until or beyond 2033. These contractual arrangements influence decision-making procedures at both local and national governments. This is manifested by the exclusion of waste incineration from the UK ETS, a decision that was made due to the argument that it would negatively impact the economic viability of WtE processes mid-point of their contracts and increase the financial burden to local authorities, which could, in turn, lead to a disruption in the waste management chain. This has ultimately driven the adoption of the WtE option for the management of waste under the disguise of eliminating unintended consequences, caused by the disruption of waste management processes that could revert back to a diversion of waste to landfill.

Binding in long-term incineration contracts creates demand for a continuous residual waste input (often of a certain composition), and this is likely to slow down residual waste reduction and its sustainable management in the long term. The commissioning of expansion projects of existing WtE plants such as the ‘Riverside 2’, which is set to be operational in 2026 (Cory, n.d.), is an indication that a reduction in residual waste generation and improvements in its management are pre-destined to fail. Increasing WtE capacity in London may be vital to divert waste from landfill for good. However, this should not be used as a misguided attempt to promote WtE, but rather as a way to place a greater focus on reducing residual waste generation. Making improvements in the segregation of waste generated in the household, could contribute to improvements in recycling and reuse rates. If the segregation and management of waste fail to improve, this will devalue efforts to promote circularity.

However, circularity is not synonymous with sustainability, and notably, residual waste is an inevitable artefact of our needs and modern lifestyles. Some types of wastes generated (e.g. sanitary, recyclables that are heavily damaged, miscellaneous materials) cannot be recycled, nor reused, in an environmentally safe, cost-efficient, socially acceptable, and technologically viable manner. Hence, treating these wastes via incineration with energy recovery, can guarantee the capture of some of their embedded multidimensional value, leading to positive impacts. Trade-offs are present in every resource and waste management system, therefore, the ability to view and orchestrate residual waste management in a way that returns the maximum net positive value whilst accommodating potential trade-offs is of the utmost importance. To achieve that one must grow the ability to view resource and waste management via the lens of a systems-based approach to examine and understand the needs of the system as a whole, identify points to intervene and make an effort to introduce changes and adopt behaviours that can deliver maximum environmental, economic, social and technical value (Iacovidou et al., 2020). Moreover, there are multiple stakeholders that influence and can be influenced by changes in residual waste amounts and composition; greater insight into these power dynamics is needed to understand the feasibility of reducing and improving the management of residual waste and moving the management of waste higher up in the waste hierarchy (Iacovidou et al., 2020). In this context, transparency and improved communication among all stakeholders are key to creating a level playing field for changes to be made gradually and at a pace that allows for risks to be managed, hence allowing stable and attainable progress to be made.

## Conclusions

Residual waste is a key fraction of the total MSW generated containing mixed wastes, which has been receiving little attention over the past years. Efforts to promote resource efficiency in the waste management sector have now placed attention on residual waste management. The initial stage to growing an understanding of this waste stream is to explore its potential fate to identify weaknesses and formulate a plan that would allow sustainability improvement in its management. In this endeavour, the definition of the residual waste stream should be agreed upon within the narrative of the circular economy, to allow for improved communication across policy and industry levels, and the development of a transparent platform for data recording and monitoring. Currently, existing data that is used to support decision- and policy-making are obscure and create many blindspots in the waste management chain, hence weakening the effect of any policy intervention. Furthermore, the contractual arrangement of local governments with waste management providers has created a reliance on specific treatment options, such as WtE facilities. On the one hand, this has led to a technological lock-in that could jeopardise the UK’s ambition and commitment to reducing its greenhouse gas emissions by 78% and 100% from 1990 levels by 2033 and 2050, respectively. On the other hand, recovery treatment processes can return mutlidimensional value in the system when used to treat wastes that have no recycling or reuse ability. Clearly, there is a need to adopt a systems approach to examine residual waste management in London and across England and the UK, and identify points to intervene and gradually reduce the reliance on WtE and landfilling and intensify efforts to promote improved segregation of waste at source. This will not only eliminate residual waste generation and mismanagement, it will also improve waste quality to potentially drive the management of waste higher in the waste hierarchy.

### Supplementary Information

Below is the link to the electronic supplementary material.

Supplementary file1 (DOCX 96 KB)

## Data Availability

The data that support the findings of this study are publicly available on the Waste Data Interrogator—Wastes Received (Excel)- Version 4, published—01/02/2022: https://environment.data.gov.uk/portalstg/home/item.html?id=f4adcd438cb144f8ad2b24529bbec78f.
