# Supplementary material for: Residual waste management in London, England: a reality check
Source: Environ Monit Assess. 2023 Oct 9;195(11):1283. doi: 10.1007/s10661-023-11760-2 (PMC10562262; doi:10.1007/s10661-023-11760-2)
Supplement: Supplementary file 1 — Supplementary file1 (DOCX 96 KB) [file 10661_2023_11760_MOESM1_ESM.docx]

# SUPPLEMENTARY MATERIAL

**Residual Waste Management in London, England: A Reality Check**

Nadia Minhas^a^ and Eleni Iacovidou^a,*^

*^a^* *Division of Environmental Sciences, College of Health, Medicine and Life Sciences, Brunel University London, Uxbridge, UB8 3PH, United Kingdom*

*^*^Corresponding Authors: Eleni Iacovidou,* [*eleni.iacovidou@brunel.ac.uk*](mailto:eleni.iacovidou@brunel.ac.uk)*, telephone: +44(0) 1895 266122*

This document provides important insights into the data analysed, the D (Disposal) and Recovery (R) codes used to distinguish between waste treatment options and provides granularity in the data collected and analysed.

# S1: London Boroughs and their population

**Table S1.** London Boroughs with their population based on census data 2021. Census data was chosen as data for 2020 were estimated.

| **London Borough** | **The population as per census data 2021** |
| --- | --- |
| Camden | 210,100 |
| City of London | 8,600 |
| Hackney | 259,200 |
| Hammersmith and Fulham | 183,200 |
| Haringey | 264,200 |
| Islington | 216,600 |
| Kensington and Chelsea | 143,400 |
| Lambeth | 317,600 |
| Lewisham | 300,600 |
| Newham | 351,100 |
| Southwark | 307,700 |
| Tower Hamlets | 310,300 |
| Wandsworth | 327,500 |
| Westminster | 204,300 |
| Barking and Dagenham | 218,900 |
| Barnet | 389,300 |
| Bexley | 246,500 |
| Brent | 339,800 |
| Bromley | 330,000 |
| Croydon | 390,800 |
| Ealing | 367,100 |
| Enfield | 330,000 |
| Greenwich | 289,100 |
| Harrow | 261,300 |
| Havering | 262,000 |
| Hillingdon | 305,900 |
| Hounslow | 288,200 |
| Kingston upon Thames | 168,000 |
| Merton | 215,200 |
| Redbridge | 310,300 |
| Richmond upon Thames | 195,200 |
| Sutton | 209,600 |
| Waltham Forest | 278,400 |

**London borough population estimates - 2021 :**

<https://www.ons.gov.uk/peoplepopulationandcommunity/populationandmigration/populationestimates/datasets/populationandhouseholdestimatesenglandandwalescensus2021>

# S2: D&R codes NAVIGATION

**Table S2.** D&R operations codes. Adopted from the Environment Agency- (D and R flow chart) and the Waste Framework Directive (WFD) Annexe I and II.

| **Codes** | **Description** | **Sub-category** | **Description** |
| --- | --- | --- | --- |
| **D01** | Landfill | D01.01 | Inert waste landfill |
|  |  | D01.02 | Non-hazardous waste |
| **D02** | Land treatment without agricultural benefit or improvement | n/a | ‘Land treatment (e.g. biodegradation of liquid or sludgy discards in soils, etc.)’ |
| **D03** | Deep injection for disposal | n/a | ‘Deep injection (e.g. injection of pumpable discards into wells, salt domes or naturally occurring repositories, etc.)’ |
| **D04** | ‘Surface impoundment’ | n/a | (e.g. placement of liquid or sludgy discards into pits, ponds or lagoons, etc.)’ |
| **D05** | Landfill | n/a | Specially engineered landfill |
| **D06** | ‘Release into a water body except for seas/oceans’ | n/a |  |
| **D07** | ‘Release to seas/oceans including sea-bed insertion’ | n/a |  |
| **D08** | Treatment prior to disposal | D08 | Biological treatment for the purpose of disposal |
|  |  | D08.01 | Mechanical biological treatment |
| **D09** | Physical/Physico-chemical treatment | n/a | Physical/ Physico-chemical treatment prior to disposal |
| **D10** | Incineration on land for the purpose of disposal | D10.01 | Municipal waste incineration |
| **D11** | ‘Incineration at sea’ | n/a |  |
| **D12** | ‘Permanent storage | n/a | (e.g. emplacement of containers in a mine, etc.)’ |
| **D13** | Blending or mixing prior to disposal | n/a | ‘Blending or mixing prior to submission to any of the operations numbered D 1 to D 12’. |
| **D14** | Transfer for disposal | n/a | ‘Repackaging prior to submission to any of the operations numbered D1 to D13’ |
| **D15** | Storage for disposal | n/a | The temporary storage of waste pending any operations D1-D14. |
| **R01** | Incineration of waste for use principally as a fuel or other means to generate energy | R01.03 | Co incineration of Refuse derived fuel (RDF) and other waste in power stations and incineration plants |
| **R02** | Solvent recovery | n/a | Solvent reclamation/regeneration |
| **R03** | “Recycling/reclamation of organic substances which are not used as solvents (including composting and other biological transformation processes)” | R03.01  R03.01.01 | Sorting organic wastes for recovery - paper |
|  |  | R03.01.02 | Sorting organic wastes for recovery – plastic |
|  |  | R03.01.04 | Sorting organic wastes for recovery – green/garden waste |
|  |  | R03.01.06 | Sorting organic wastes for recovery – clothing/textiles |
|  |  | R03.01.07 | Sorting organic wastes for recovery – wood/plastic/textiles |
|  |  | R03.03 | Anaerobic digestion (green and kitchen waste) |
|  |  | R03.04 | Mechanical Processing |
|  |  | R03.04.02 | Mechanical reprocessing of wood (for recycling/energy recovery) |
|  |  | R03.04.03 | Mechanical reprocessing for production of refuse derived fuel |
|  |  | R03.06 | Mechanical – physical stabilisation |
| **R04** | Recycling/reclamation of metals and metal compounds | R04.01.01 | Bulking up metals- metal packaging i.e. cans |
|  |  | R04.02 | Mechanical processing |
| **R05** | Recycling/reclamation of other inorganic materials | R05.01 | Bulking up glass |
|  |  | R05.03.02 | Mechanical reprocessing of mixed C&D waste |
|  |  | R05.06.01 | Manufacture of new products from inorganic waste (Recycling of glass) |
|  |  | R05.06.02 | Manufacture of new products from inorganic waste (Manufacture of building materials.) |
| **R06** | Regeneration of acids and bases | n/a |  |
| **R07** | Recovery of pollution abatement components |  |  |
| **R08** | Recovery of components from catalysts | n/a |  |
| **R09** | Oil refining or reuse of waste oil | n/a |  |
| **R10** | Land treatment resulting in benefit to agriculture or ecological improvement | n/a |  |
| **R11** | Use of waste | n/a | Use of waste obtained from any of the operations numbered R 1 to R 10 |
| **R12** | Exchange of waste for submission to any of the operations numbered R 1 to R 11 | n/a | Exchange of waste for submission to any other recovery operation R1-R10 |
| **R13** | Temporary storage | n/a | Temporary storage of wastes pending any other recovery operation (excluding temporary storage, pending collection, on the site where it is produced). |

**Key links:**

- Full Disposal and Recovery Flow chart - Environment Agency: <http://www.wastesupport.co.uk/recovery-and-disposal-codes/>
- Waste Framework Directive Annex I: <https://www.legislation.gov.uk/eudr/2008/98/annex/I>
- Waste Framework Directive Annex II: <https://www.legislation.gov.uk/eudr/2008/98/annex/II>
- 2020 Waste Data Interrogator - Wastes Received (Excel)- Version 4, published - *01/02/2022*:

<https://environment.data.gov.uk/portalstg/home/item.html?id=f4adcd438cb144f8ad2b24529bbec78f>

- The EWC-Stat categories are: <https://ec.europa.eu/eurostat/documents/342366/351806/Guidance-on-EWCStat-categories-2010.pdf/0e7cd3fc-c05c-47a7-818f-1c2421e55604>
- List of Waste codes: <https://www.legislation.gov.uk/uksi/2005/895/schedule/1/made>
- Waste contract register: <https://data.london.gov.uk/dataset/waste-contracts-register>

# S3: Residual waste reporting in WDI and WDF

The following Tables present detailed information on residual waste generation across all 32 boroughs in London from two databases; the Waste Data Interrogator (WDI) and the Waste Data Flow (WDF). It also presents evidence on the fate of residual waste according to the D&R codes used by waste management professionals.

**Table S3. Residual waste tonnage reporting for London boroughs from the WDI and WDF**

| **WDA/UA** | **Authority** | **WDF**  **(Household)** | **WDF**  **(Non-Household)** | **WDF**  **(Combined)** | **WDI** |
| --- | --- | --- | --- | --- | --- |
| NLWA | **Barnet** | 99,239.47 | 11,677.87 | 110,917.34 | 145,751.1 |
| NLWA | **Enfield** | 68,368.02 | 20,333.56 | 88,701.58 | 42,580.6 |
| NLWA | **Haringey** | 56,993.84 | 17,493.14 | 74,486.98 | 24,883.99 |
| NLWA | **Camden** | 35,029.35 | 31,114.36 | 66,143.71 | 79,221.19 |
| NLWA | **Islington** | 36,625.09 | 36,428.65 | 73,053.74 | 105,095.4 |
| NLWA | **Hackney** | 63,200.31 | 22,901.57 | 86,101.88 | 52,663.37 |
| NLWA | **Waltham Forest** | 63,731.94 | 7,661.34 | 71,393.28 | 25,968.67 |
| ELWA | **B and D** | 71,511.21 | 4,943.19 | 76,454.4 | 138,842.9 |
| ELWA | **Havering** | 77,092.09 | 3,352.63 | 80,444.72 | 107,033.1 |
| ELWA | **Redbridge** | 83,599.17 | 5,960.72 | 89,559.89 | 108,130.7 |
| ELWA | **Newham** | 104,798.91 | 7,738.98 | 112,537.89 | 134,879.5 |
| WLWA | **Brent** | 63,387.83 | 11,242.43 | 74,630.26 | 186,838.3 |
| WLWA | **Hillingdon** | 63,779 | 18,686 | 82,465 | 142,702.8 |
| WLWA | **Harrow** | 55,924.54 | 4,102.56 | 60,027.1 | 80,768.19 |
| WLWA | **Ealing** | 43,530.27 | 34,221.33 | 77,751.6 | 138,330.9 |
| WLWA | **Hounslow** | 59,302.08 | 10,735.41 | 70,037.49 | 80,360.84 |
| WLWA | **R U T** | 45,221.29 | 4,409.55 | 49,630.85 | 66,897.08 |
| WRWA | **K and C** | 36,069.74 | 14,038.22 | 50,107.96 | 11,385.24 |
| WRWA | **H and F** | 31,911.18 | 24,286.36 | 56,197.54 | 13,000.51 |
| WRWA | **Lambeth** | 47,134.55 | 42,105.43 | 89,239.98 | 21,,237.64 |
| WRWA | **Wandsworth** | 75,126.93 | 6,865.03 | 81,991.96 | 31,573.58 |
| U-SLWP | **Croydon** | 68,975.64 | 31,973.47 | 100,949.11 | 52,228.35 |
| U-SLWP | **K U T** | 31,344.72 | 2,806.06 | 34,150.79 | 52,080.11 |
| U-SLWP | **Merton** | 41,577.21 | 6,888.65 | 48,465.86 | 10,345.52 |
| U-SLWP | **Sutton** | 38,824.75 | 3,404.95 | 42,229.7 | 4,695.74 |
| U-SELJWPG | **Greenwich** | 71,098.61 | 9,218.71 | 80,317.32 | 148,800.3 |
| U-SELJWPG | **City of London** | 2,282.49 | 866.5 | 3,148.99 | 37,463.23 |
| U-SELJWPG | **Bromley** | 68,802.19 | 19,226.22 | 88,028.41 | 172,820.6 |
| U-SELJWPG | **Southwark** | 81,395.21 | 5,855.86 | 87,251.07 | 109,370.6 |
| U-SELJWPG | **Lewisham** | 67,593.84 | 16,560.45 | 84,154.29 | 119,260.5 |
| U-SELJWPG | **Bexley** | 47,782.21 | 9,269.78 | 57,051.99 | 77,201.48 |
| Unitary | **Westminster** | 46,624.78 | 55,452.53 | 102,077.31 | 106,422.4 |
| Unitary | **Tower Hamlets** | 71,674.38 | 20,842.47 | 92,516.85 | 20,780.8 |

*ELWA - East London Waste Authority; NLWA - North London Waste Authority; WLWA - West London Waste Authority; WRWA - Western Riverside Waste Authority. Unitary authorities include U-SLWP - South London Waste Partnership; U-SELJWPG - South East London Joint Waste Planning Group; U- Unitary authorities, that do not belong to a non-statutory group/partnership.*

# S4: rESIDUAL WASTE fate

**Figure S4.1 Total residual waste tonnage (kt) under the London variation, namely, ‘London’, ‘Central London’, and ‘Greater London’. The total residual waste reported for all London variations is 2.44 Mt. Residual waste calculated for the London variation is 1.89 Mt tonnes. Greater London is associated with 545.38 kt of residual waste. Central London reported 1.30 kt of residual waste.**

**Figure S4.2 Total amount of residual waste reported in recovery and disposal operations in London, 2020. Total residual waste reported under recovery operations is 3,491,898 tonnes and disposal operations is 1,590,129.**

**Figure S4.3 Total residual waste with D and R codes composition.**

# S5: Residual waste wdi Granular r sub-codes

**Figure S5.1 All sub-codes reported under code R01 in (kt). R01: 1,506,545 and R01.03 10,683.87 (tonnes)**

**Figure S5.2 All sub-codes reported under code R03 in (kt): R03 61,645.6; R03.01: 222,610; R03.04.03: 28,775.29; R03.01.01: 23,373.4; R03.04: 6,404.02; R03.03: 6,201; R03.06: 1888.72; R03.01.02: 735.6; R03.01.06: 445.96; R03.01.07: 250.82; R03.04.02: 312.68; R03.02.04: 1.28 (tonnes).**

**
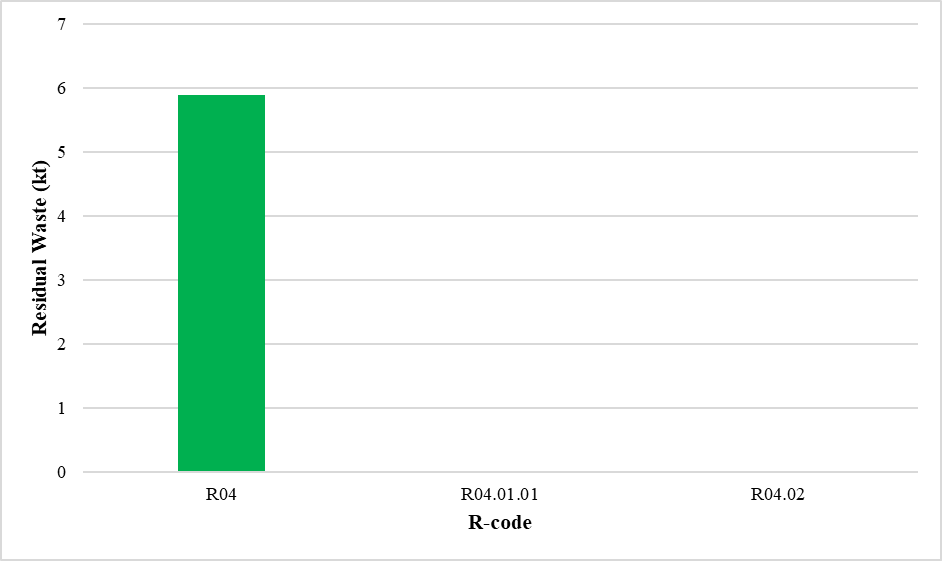
**

**Figure S5.3All sub-codes reported under code R04 in (kt). R04: 5896.91 R04.01.01: 3.3 R04.02: 3.44 (tonnes).**

**Figure S5.4 All sub-codes reported under code R05 in (kt): R05: 76,000; R05.01: 30,524.17; R05.03.02: 4,436.28; R05.06.01: 74.6; R05.06.02: 48 tonnes.**

# S6: residual waste wdi - granular d sub codes

**Figure S6.1 All sub-codes reported under code D01 in (kt). D01.01: 23,771.43; D01.02: 7,645.34; D01: 1,500.44 tonnes.**

**Figure S6.2 All sub-codes reported under code D08 in (kt). D08: 309,405.9; D08.01: 69,926.52 tonnes.**

**Figure S6.3 All sub-codes reported under code D10 in (kt). D10: 565,732.1 D10.01: 7,572.94 (tonnes).**
